# Laying the Foundation-Stone

**Published:** 1920-10-23

**Authors:** 


### THE BRITISH HOSPITAL FOR MOTHERS AND BABIES.

### Laying the Foundation-stone.

On Saturday afternoon Princess Christian, attended by Miss Lock and Mr. Hugo Wemyss, visited Woolwich, in beautiful sunshine, to lay the foundation-stone of the new building of the British Hospital for Mothers and Babies, which is also to be a national training-school for midwives. She was received by Sir Richard Temple (the President), Dr. Wise, Mr. Mary Blair, Miss Alice Gregory (the Hon. Secretary), and Mrs. Parnell (matron of the home). There were present Major-General Sir John and Lady Longhey, Colonel and Lady Edwina Lewin, and the Countess of Stamford—whose daughter, Lady Jane Grey, presented her Royal Highness with a bouquet of pink carnations—and the Mayor and Mayoress of Woolwich. The new building will cost about £100,000, and will stand in three acres of grounds, on an admirable site. Already the wards have been begun. It was feared at one time that the funds in hand would not allow of any other work being started. But on Saturday Sir Richard Temple, who is President of the Hospital, was able to make the welcome announcement that the Charity Commissioners had given her Royal Highness £8,000 for the scheme, and that the Minister of Health had promised £20,000. This will permit of the administration block being put in hand at once. It is hoped later that an ante-natal and pre-natal department will be added.

The ceremony partook largely of a religious character, the Bishop of Woolwich being present, as well as several clergy and a large choir. The various local industries were splendidly represented. Purses, each containing £10, were handed to Princess Christian by small children on behalf of numerous factories, firms, and streets in the neighbourhood. They marched in procession in groups of three, carrying banners, the details in every way being excellently arranged. After the presentation of purses, Old English dances were given by scholars, many of very tender years, from the various London County Council schools, which Princess Christian watched with much interest. It is hoped that the buildings may be completed in the course of next year, when there will be accommodation for forty mothers. The present home can only receive eighteen, and the beds could be filled several times over, so great is the demand.

